# Formation of Drug-Participating Catanionic Aggregates for Extended Delivery of Non-Steroidal Anti-Inflammatory Drugs from Contact Lenses

**DOI:** 10.3390/biom9100593

**Published:** 2019-10-10

**Authors:** Cesar Torres-Luna, Abdollah Koolivand, Xin Fan, Niti R. Agrawal, Naiping Hu, Yuli Zhu, Roman Domszy, Robert M. Briber, Nam Sun Wang, Arthur Yang

**Affiliations:** 1 Department of Chemical & Biomolecular Engineering, University of Maryland, College Park, MD 20740, USA; cesartorres093@gmail.com (C.T.-L.); kolivand@umd.edu (A.K.); nitiagr@umd.edu (N.R.A.); dwillyu@163.com (Y.Z.); 2 Department of Chemical Engineering, Auburn University, Auburn, AL 36849, USA; xzf0004@tigermail.auburn.edu; 3 Lynthera Corporation, 1200 Corporate Blvd., STE 10C, Lancaster, PA 17601, USA; naiping.hu@gmail.com (N.H.); rcdom@istninc.com (R.D.); 4 Department of Materials Science and Engineering, University of Maryland, College Park, MD 20740, USA; rbriber@umd.edu

**Keywords:** drug delivery, contact lenses, controlled release, cationic surfactant, aggregates, rheology, NSAID, wormlike micelles, Maxwell model, hydrogel

## Abstract

This paper focuses on extending drug release duration from contact lenses by incorporating catanionic aggregates. The aggregates consist of a long-chain cationic surfactant, i.e., cetalkonium chloride (CKC), and an oppositely charged anti-inflammatory amphiphilic drug. We studied three non-steroidal anti-inflammatory (NSAID) drugs with different octanol–water partition coefficients; diclofenac sodium (DFNa), flurbiprofen sodium (FBNa), and naproxen sodium (NPNa). Confirmation of catanionic aggregate formation in solution was determined by steady and dynamic shear rheology measurements. We observed the increased viscosity, shear thinning, and viscoelastic behavior characteristic of wormlike micelles; the rheological data are reasonably well described using a Maxwellian fluid model with a single relaxation time. In vitro release experiments demonstrated that the extension in the drug release time is dependent on the ability of a drug to form viscoelastic catanionic aggregates. Such aggregates retard the diffusive transport of drug molecules from the contact lenses. Our study revealed that the release kinetics depends on the CKC concentration and the alkyl chain length of the cationic surfactant. We demonstrated that more hydrophobic drugs such as diclofenac sodium show a more extended release than less hydrophobic drugs such as naproxen sodium.

## 1. Introduction

Ocular drug delivery is a challenging research field due to the presence of anatomical and physiological barriers that affect the drug bioavailability following multiple routes of administration, including topical, systemic, and injectable [1]. Most ocular diseases are treated with topical application of eye drops, which account for nearly 90% of the currently accessible marketed formulations [2]. However, eye drops are inefficient, suffering from tear drainage, in addition to corneal and sclera barriers. Only about 5% of functional ingredients can be delivered as a burst dosage [3,4]. In addition, frequent dosing schedules often decrease patients’ adherence to the treatment regimen for chronic eye diseases.

In order to address the limitations of eye drops, researchers have explored the use of therapeutic contact lenses [5,6]. When contact lenses containing ophthalmic drugs are placed on the eye, the drug diffuses through the lens matrix and enters the post-lens tear film, in which drug molecules have a longer residence time in comparison to eye drops [7]. Nevertheless, a major limitation of unmodified contact lenses is that most of the drug is released within the first few hours [8]. Several methods have recently been employed to increase the release duration of drugs from contact lenses: nanoparticle-based contact lenses [9,10,11,12,13,14], biomimetic and imprinted contact lenses [15], vitamin *E* hydrophobic coating [3,4,16,17,18,19,20], and layer-structured contact lenses [21]. A limited number of these approaches have been examined in clinical trials, such as the treatment of ocular allergy with ketotifen [22] and the treatment of glaucoma with timolol and dorzolamide [23].

The use of catanionic aggregates formed from drug compounds with oppositely-charged surfactants has received particular attention for drug delivery applications [24,25,26,27,28,29,30,31,32,33]. Aggregates made of ionic drugs and surfactants have proven to extend the drug release from synthetic gels from hours to days due to the presence of vesicles or entangled micelles [24,26]. Wormlike micelles are known to have beneficial immunotherapeutic effects due to their elongated shapes, which provide high surface area and large aspect ratio [34]. Recently, a drug-participating catanionic system has been developed for effective delivery of the chemotherapeutic agent chlorambucil [35]. This drug, containing catanionic aggregate system, was demonstrated to be a safe and efficient drug delivery system that can be used for cancer therapy [35].

Researchers have used micelles made of non-ionic surfactants in contact lenses to extend the delivery time of hydrophobic drugs such as cyclosporine A [12,36,37], dexamethasone [37], and dexamethasone 21-acetate [37]. Micelles made of non-ionic surfactants only extended the release of cyclosporine A [12,36,37]. For dexamethasone and dexamethasone 21-acetate, no extended release was observed due to the insufficient partition of the drugs in the surfactant aggregates [37]. Chauhan et al. [38] developed a method to extend the delivery of an anionic drug from contact lenses through the use of a long-chain cationic surfactant. The release of dexamethasone 21 di-sodium phosphate increased from 2 to 50 h due to electrostatic interactions between the positively-charged contact lens and the negatively-charged drug. However, it was explained that the release kinetics was improved only due to charge interactions and not due to the formation of ionic surfactant aggregates [38].

In this paper, we report the preparation of aggregates composed of a cationic surfactant and an anionic drug for the purpose of extending drug delivery from poly-hydroxy-ethyl-methacrylate (pHEMA) contact lenses. We study non-steroidal anti-inflammatory drugs (NSAIDs), a class of drugs that is used for ocular inflammation and pain relief [39]. Due to the amphiphilic nature and the physiological negative charge of NSAIDs [40,41], our approach is based on the idea that these drugs can act as strong binding counterions and form catanionic aggregates with long-chain cationic surfactants. As a result, the entrapment of the drug molecules in the aggregates leads to the extension of the release time from contact lenses. We characterize the viscoelastic behavior of the aggregates using steady and dynamic shear rheology. We also discuss the in vitro release kinetics of contact lenses and assess the impact of the aggregates on extending drug release.

## 2. Materials and Methods

### 2.1. Materials

Diclofenac sodium (DFNa), naproxen sodium (NPNa), Brij 97 (B97), cetalkonium chloride (CKC), 2-hydroxyethyl methacrylate (HEMA), ethylene glycol dimethacrylate (EGDMA), azobis-*iso*-butrylonitrile (AIBN), benzyl-dimethyl-octyl-ammonium chloride (C8), and Dulbecco’s phosphate buffer saline (PBS) were purchased from Millipore-Sigma (St. Louis, MO, USA). Flurbiprofen sodium (FBNa) reference standard was purchased directly from the United States Pharmacopeia (USP, Rockville, MD, USA). All chemicals were used as received.

### 2.2. Preparation of Drug–Surfactant Solutions

We obtained homogeneous surfactant solutions by mixing 2 g of Brij 97 in 10 mL of deionized water with 0, 125, 250, 500, 750, or 1000 mg of CKC. The amounts of CKC correspond to concentrations of 0, 25, 50, 100, 150, and 200 mM CKC, respectively. Because CKC has limited water solubility, a non-ionic surfactant was used to increase its aqueous solubility. We stirred each solution at 800 rpm at room temperature until the CKC was completely dissolved. Next, 50 mg of drug (DFNa or NPNa) was dissolved with 5 mL of the prepared surfactant solution. For FBNa, 25 mg was used instead of 50 mg. Drugs were dissolved for 20 min at 600 rpm at room temperature.

### 2.3. Rheological Studies of Drug–Surfactant Solutions

Steady and dynamic shear rate rheological experiments were performed on an AR2000 controlled stress rheometer (TA Instruments, Newark, DE, USA) using cone-and-plate geometry (20 mm diameter, 2° cone angle). Dynamic frequency spectra were conducted in the linear viscoelastic regime of the solutions, as determined from dynamic stress sweep measurements.

### 2.4. Fabrication of Contact Lenses

We fabricated the contact lenses by thermally curing the monomer solutions in polypropylene lens molds. The HEMA monomer (2 mL) was mixed with 4.5 mg AIBN as thermal polymerization initiator, 7.5 μL EGDMA as crosslinker, and 1.5 g drug–surfactant solution. This solution was stirred at 300 rpm at room temperature for 10 min and three drops (~75 μL) of the mixed solution was poured into the contact lens mold (14 mm diameter, 8.4 mm base curve radius, and 120 μm thickness). After curing at 70 °C for 24 h and demolding, the contact lenses were thoroughly washed in deionized water for 1 h to remove unreacted monomers.

### 2.5. In Vitro Release Experiments

Each contact lens was immersed in 3 mL of PBS at pH 7.4 and room temperature. At predetermined time intervals, 1 mL aliquots were pipetted out and replaced by 1 mL fresh PBS. The drug concentration was determined using a ultraviolet (UV)-visible spectrophotometer (Varian Cary 50 Bio, Walnut Creek, CA, USA) at wavelengths of 276 nm for DFNa, 248 nm for FBNa, and 271 nm for NPNa. Control contact lenses (i.e., with no drug) were tested in the in vitro release experiments to ensure that there were no interfering absorbance readings.

### 2.6. Drug Extraction from Contact Lenses

To determine the total amount of drug in the contact lenses, we immersed each lens in 3 mL of ethanol for 24 h to extract all of the drug. The total amount of drug per each lens was quantified using a UV-visible spectrophotometer (Varian Cary 50 Bio) with pre-established calibration curves. After the extraction, lenses were discarded and not used in subsequent experiments.

### 2.7. Characterization of Contact Lenses

#### 2.7.1. Transmittance Analysis

The optical transparency was measured using a UV-visible spectrophotometer (SpectraMax i3, Molecular Devices, Sunnyvale, CA, USA) from 380 to 740 nm wavelength range. Contact lenses were hydrated overnight by soaking in PBS at pH 7.4, and they were placed in a 24-well plate filled with 1 mL of PBS to measure transmittance.

#### 2.7.2. Water Content

The water content of contact lenses was conducted by immersion of each contact lens in 2 mL of deionized water at room temperature for 24 h. The contact lens was removed from the solution, gently blotted to remove all the liquid from the surface, and wet lens weight was recorded. Dry weights were measured after removing the contact lenses from the hydrating solution and letting them dry at room temperature overnight. The following equation was used to calculate the % water content of contact lenses:

(1) $$Water\;content\;\left(\%\right)=\frac{Weight_{wet}-Weight_{dry}}{Weight_{wet}}\times 100.$$

## 3. Results and Discussion

### 3.1. Steady and Dynamic Shear Rheology

**Figure 1:**
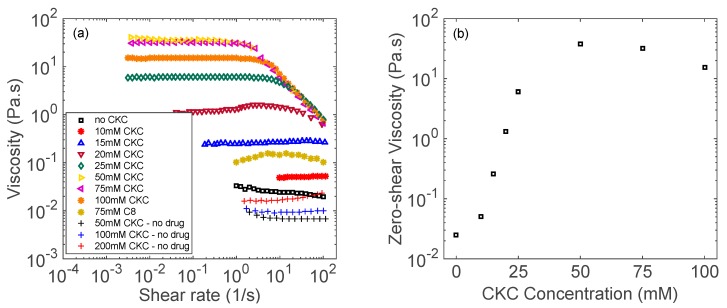
(**a**) Shear viscosity as a function of shear rate for aggregates made of cetalkonium chloride (CKC) and diclofenac sodium (DFNa). (**b**) Zero-shear viscosity as a function of CKC concentration. Measurements were performed at room temperature and at a fixed concentration of diclofenac sodium (30 mM) and Brij 97 (20 wt%).

Figure 1a shows the steady-shear rheology of catanionic aggregates between DFNa and cetalkonium chloride (CKC). Below 15 mM CKC, solutions behave as Newtonian fluids with relatively low values of viscosity. At 20 mM CKC, non-Newtonian behavior is observed at shear rates above 10 s^−1^. For concentrations of 25 mM CKC or higher, shear thinning behavior with a plateau in the viscosity at low shear rates is observed for all systems. Furthermore, unlike CKC, C8 is unable to aggregate with DFNa even at high concentrations, as demonstrated by its relatively low solution viscosity. As shown in Figure 1a, 75 mM C8 solution behaves as a Newtonian fluid with viscosity lower than the 15 mM CKC solution.

In addition, from Figure 1b, 10 mM CKC aggregates have a zero-shear viscosity of 0.05 Pa.s., while 25 mM CKC aggregates have a zero-shear viscosity of 6.0 Pa.s. The increase in CKC concentration from 10 to 25 mM, results in a significant increase in zero-shear viscosity by more than two orders of magnitude, where the transition from Newtonian to non-Newtonian behavior occurs. Shear thinning behavior [42] and the notable increase in viscosity [24,26,30] can be associated to the presence of elongated wormlike micelles in solution. Other rheological studies have also reported a similar behavior [43,44]. Raghavan et al. [43] conducted a study of wormlike micelles between a long-chain cationic surfactant and sodium salicylate as the binding salt. It was reported that by increasing the surfactant concentration at a fixed sodium salicylate concentration, the zero-shear viscosity had an exponential increase and then leveled off at higher surfactant concentrations [43]. Kim et al. [44] studied wormlike micelles from cetyltrimethylammonium bromide (CTAB) and sodium salicylate, reporting that the shear viscosity of the wormlike micelles increased abruptly at a certain CTAB concentration near 10 mM.

**Figure 2:**
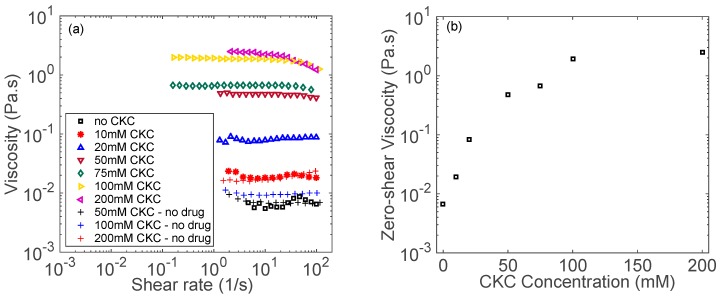
(**a**) Shear viscosity as a function of shear rate for aggregates made of cetalkonium chloride (CKC) and naproxen sodium (NPNa). (**b**) Zero-shear viscosity as a function of CKC concentration. Measurements were performed at room temperature and at a fixed concentration of naproxen sodium (30 mM) and Brij 97 (20 wt%).

Figure 2 shows the steady-shear rheology of aggregates between NPNa and CKC. Solutions at 75 mM CKC and below behave as Newtonian fluids that are independent of shear rate. At 100 mM CKC and above, a shear thinning behavior is observed above a shear rate of 10 s^−1^. However, 100 mM and 200 mM CKC solutions with NPNa exhibit a very weak shear thinning behavior. From Figure 2, for the 100 mM CKC solution with NPNa, the viscosity decreases from 2 Pa.s at a shear rate of 1 s^−1^, to 1 Pa.s at a shear rate of 100 s^−1^. For DFNa at 100 mM CKC (Figure 1a), the viscosity decreases from 15 Pa.s at a shear rate of 1 s^−1^, to 1 Pa.s at a shear rate of 100 s^−1^. Owing to the stronger shear thinning behavior of the DFNa solution, its viscosity decreases by a factor of 15, while for the NPNa solution, we observe a smaller decrease by a factor of 2.

It is worth to note that for the control samples containing only CKC (50, 100, and 200 mM) with no drug, no shear thinning behavior is observed, as shown in Figure 1a and Figure 2a. Therefore, it can be concluded that the observed shear thinning behavior results from the interactions between the drug and CKC.

As it can be seen from Figure 1 and Figure 2, NPNa solution requires approximately five times the CKC concentration than DFNa solution in order to show a shear thinning behavior. This is because the formation of wormlike micelles depends on the molecular architecture of the aromatic counterion [45]. For instance, more hydrophobic counterions induce the formation of wormlike micelles at lower surfactant concentrations [46]. DFNa has an octanol–water partition coefficient (log *P*) of 4.75, while NPNa has a log *P* of 3.39 (see Table 1). Due to their hydrophobicity difference, it is likely that wormlike micelles form at lower CKC concentrations for DFNa than for NPNa.

**Figure 3:**
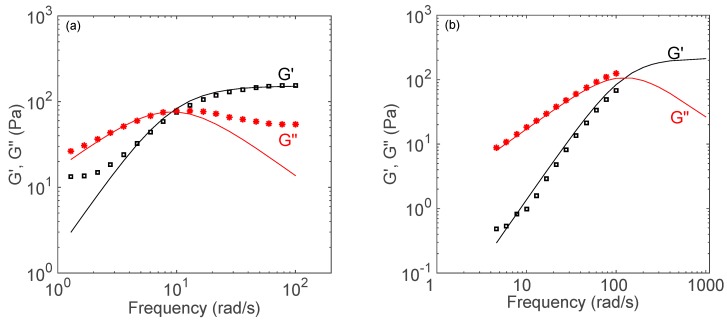
(**a**) Dynamic rheology of a 75 mM CKC sample with DFNa at 30 mM CKC. (**b**) Dynamic rheology of a 200 mM CKC sample with NPNa at 30 mM CKC. The elastic modulus G′ is shown as black unfilled squares and the viscous modulus G″ is shown as red unfilled triangles. Measurements were performed at room temperature.

The dynamic rheological response of catanionic aggregates is presented in Figure 3. The elastic *G*′ and viscous *G*″ moduli are plotted as a function of frequency *ω*. For DFNa with a CKC concentration at 75 mM, the frequency spectrum shows plateau modulus *G_p_* of 150 Pa and a crossover of *G*′ and *G*″ at approximately *ω_c_* = 10 rad/s. The relaxation time *t_R_* is obtained by taking the inverse of *ω_c_*. Therefore, the sample response can be divided into two regimes based on the relaxation time [47]. At time scales shorter than *t_R_* (i.e., for *ω* >> *ω_c_*), the response is elastic, whereas for time scales longer than *t_R_* (i.e., for *ω* << *ω_c_*), the response is viscous. This time-dependent viscoelastic response has been reported to be typical of wormlike micelles [47,48].

As shown in Figure 3, the dynamic response of DFNa-based catanionic aggregates can be fitted to a Maxwell fluid model. For a Maxwell fluid, the elastic (*G*′) and viscous (*G*″) moduli are described by the following equations [49]:

(2) $$G^{\prime }\left(\omega \right)=\frac{G_{p}\omega^{2}t_{R}^{2}}{1+\omega^{2}t_{R}^{2}},$$

(3) $$G^{\prime \prime }\left(\omega \right)=\frac{G_{p}\omega t_{R}}{1+\omega^{2}t_{R}^{2}}.$$

Furthermore, the zero-shear viscosity of a Maxwell fluid is given by [49]:

(4) $$\eta_{0}=G_{p}\;t_{R}.$$

The corresponding Maxwellian curves from Equations (2) and (3) are plotted as solid lines in Figure 3, and good agreement between our experimental data and the Maxwellian fluid model is observed. As shown in Figure 3a, the relaxation time *t_R_* and plateau modulus *G_p_* are 0.1 s and 150 Pa, respectively. The predicted zero-shear viscosity (based on Equation (4)) and measured zero-shear viscosity for DFNa solution at 75 mM CKC are 15 Pa.s and 32 Pa.s, respectively. The measured zero-shear viscosity for NPNa solution is 2 Pa.s (Figure 2b). Similarly, the relaxation time *t_R_* and plateau modulus *G_p_* for NPNa solution are 0.01 s and 200 Pa for NPNa (Figure 3b), which gives a theoretical zero-shear viscosity of 2 Pa.s.

In summary, the dynamic responses of these catanionic aggregates are predicted fairly well by the Maxwell fluid model with a single relaxation time, which is a signature of systems containing wormlike micelles [43,47,50,51]. Note that, as shown in Figure 3a (DFNa), the Maxwell model fits the data well at low and intermediate frequencies. The *G*″ data depart from the Maxwell model at high frequencies. This discrepancy is known to be linked with the presence of wormlike micelles in the solution, because micelles are dynamic entities that break and recombine rapidly [43,47,51].

### 3.2. In Vitro Release Kinetics

#### 3.2.1. Effect of the Hydrophobic Tail Length in Cationic Surfactant

**Figure 4:**
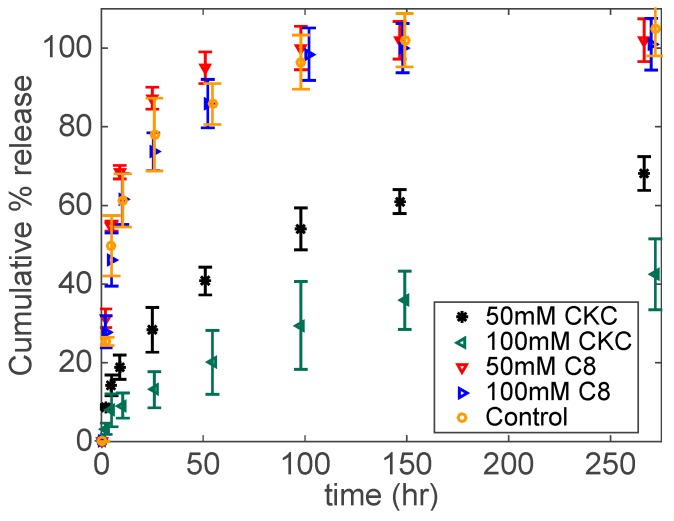
DFNa cumulative % release as a function of time under two different concentrations of CKC or C8. Catanionic aggregates were prepared at a fixed concentration of DFNa (30 mM) and Brij 97 (20 wt%). Total amount of drug per lens: 102.6 ± 9.2 μg for 50mM C8; 94.7 ± 7.6 μg for 100 mM C8; 120.0 ± 12.4 μg for 50 mM CKC; 134.3 ± 18.5 μg for 100 mM CKC. Data are presented as mean ± standard deviation with *n* = 3.

Here, we study two different derivatives of benzalkonium chloride (BKC): CKC and benzyl-di-methyl-octyl-ammonium chloride (C8). CKC is a 16-carbon-unit alkyl benzalkonium chloride derivative, while C8 is an 8-carbon-unit alkyl benzalkonium chloride derivative. BKC is an eye drop preservative used in approximately 70% of commercially available topical ophthalmic formulations [52]. Figure 4 shows the release kinetics for DFNa under two different concentrations (i.e., 50 and 100 mM CKC) for both C8 and CKC. The drug release is strongly dependent on the hydrophobic tail-length, since it is significantly slower for CKC in drug-participating catanionic systems embedded in contact lenses. For both C8-based contact lenses, 100% of DFNa is released after 100 h. On the other hand, approximately 55% and 30% of DFNa releases after 100 h for 50 mM CKC and 100 mM CKC contact lenses, respectively.

In drug-containing catanionic aggregates, cationic surfactants interact with anionic-charged drugs due to both electrostatic and hydrophobic interactions [53,54,55,56,57]. Rajput et al. [56] studied the drug-induced structural transition of catanionic aggregates made of diclofenac sodium and quaternary ammonium bromide cationic surfactants. They concluded that increasing the alkyl chain length of the cationic surfactant from twelve to sixteen carbon units required less DFNa for the micellar transition from spherical to elongated wormlike micelles and vesicles. Vaid et al. [55] concluded that the effect of diclofenac sodium on the average hydrodynamic diameter of micellar aggregates increases as a function of the length of the cationic surfactant, which indicates the importance of the hydrophobic interactions between the drug and the surfactant. In our study, the hydrocarbon chain length of C8 is half that of CKC. Consequently, we expect less hydrophobic interactions between DFNa and C8. As shown in Figure 1a, the C8 solution holds a relatively low viscosity in comparison to CKC (for the same concentration, i.e., 75 mM), indicating a higher number of catanionic aggregates in the CKC solution. The presence of such aggregates enhances the drug release extension, as shown in Figure 4. Note that the “Control” group in Figure 4 represents the drug release profile of drug-only contact lenses with no surfactant. Comparing the release profiles of C8-containing contact lenses with the Control release profile, it can be concluded that the effect of C8 on extending drug release kinetics is negligible.

#### 3.2.2. Effect of Drug Type

The octanol–water partition coefficient (log *P*) has been a conventional parameter to study the lipophilic character of drug compounds, and the correlation of lipophilicity to pharmacokinetics and pharmacodynamics [58]. In this section, we study three different NSAIDs with different log *P* to evaluate the impact of this parameter in the drug release kinetics from contact lenses.

**Figure 5:**
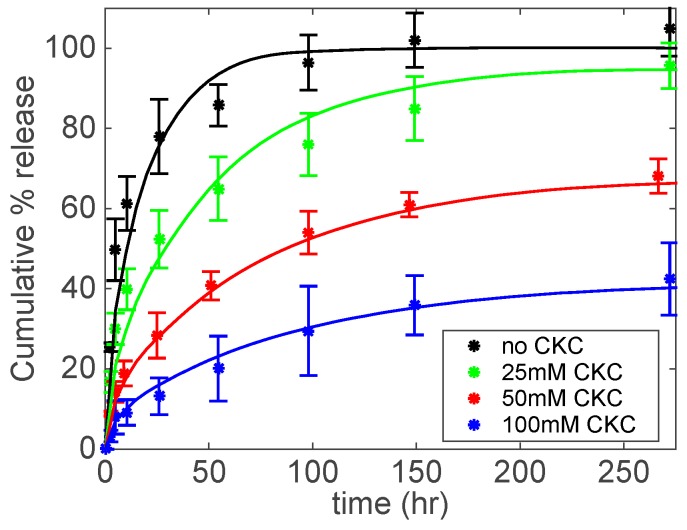
DFNa cumulative % release as a function of time under different CKC concentrations. Catanionic aggregates were prepared at a fixed concentration of DFNa (30 mM) and Brij 97 (20 wt%). Total amount of drug per lens: 119.7 ± 8.7 μg for No CKC; 142.6 ± 13.2 μg for 25 mM CKC; 120.0 ± 12.4 μg for 50 mM CKC; 134.3 ± 18.5 μg for 100 mM CKC. Data are presented as mean ± standard deviation with *n* = 3.

Figure 5 shows the release kinetics of DFNa. By increasing CKC concentration, there is a more extended release. After 100 h, control contact lenses (i.e., no CKC) have delivered almost a hundred percent of DFNa, while contact lenses with 25, 50, and 100 mM CKC released approximately 80, 55, and 30% of DFNa, respectively.

Because the drug participates in the formation of the viscoelastic catanionic systems, we speculate that the mechanism of extended release is due to the entrapment of the drug molecules in the entangled aggregates. When CKC is at low concentrations (i.e., below 25 mM CKC) or not present at all, more drug is available as “free” molecules, which is the percentage of the total drug in the gel that is neither adsorbed into the polymer matrix nor entrapped in the catanionic aggregates [37]. Most of this “free” drug is released over a short time period (e.g., <10 h). When increasing the concentration of CKC, there is less drug released during the initial period due to the presence of the catanionic aggregates in the lens matrix. The presence of aggregates inside the gel leads to an increase in the length of the path that molecules take to diffuse from inside the lens to the fluid reservoir [3].

**Figure 6:**
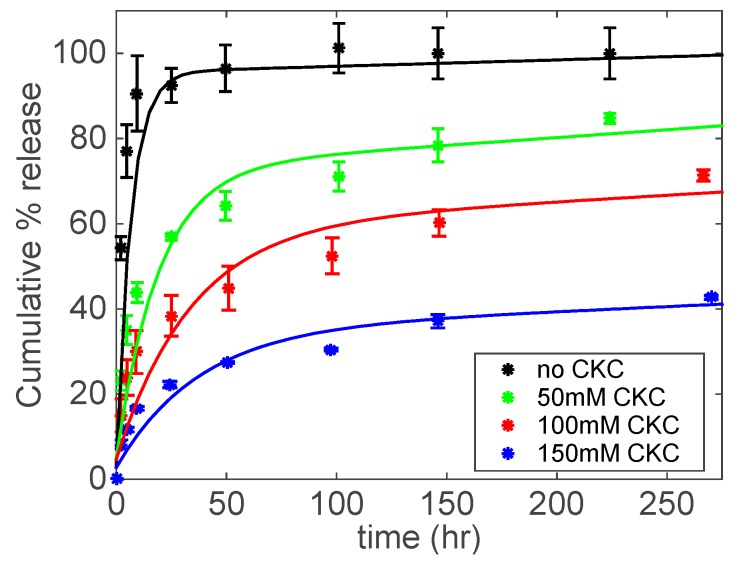
FBNa cumulative % release as a function of time under different CKC concentrations. Catanionic aggregates were prepared at a fixed concentration of FBNa (20 mM) and Brij 97 (20 wt%). Total amount of drug per lens: 77.3 ± 6.1 μg for No CKC; 88.1 ± 3.2 μg for 50 mM CKC; 69.3 ± 8.7 μg for 100 mM CKC; 83.7 ± 2.8 μg for 150 mM CKC. Data are presented as mean ± standard deviation with *n* = 3.

For FBNa, as shown in Figure 6, the control contact lenses delivered more than 90% of FBNa after 50 h. On the other hand, contact lenses with embedded catanionic aggregates at 50, 100, and 150 mM CKC released approximately 70, 45, and 25% of FBNa, respectively, after 50 h. This confirms the strong influence that the concentration of CKC has in extending drug release.

**Figure 7:**
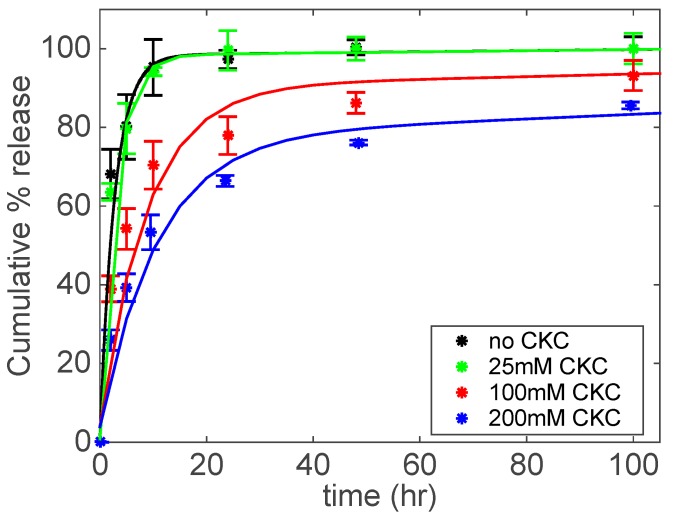
NPNa cumulative % release as a function of time under different CKC concentrations. Catanionic aggregates were prepared at a fixed concentration of NPNa (40 mM) and Brij 97 (20 wt%). Total amount of drug per lens: 106.3 ± 9.1 μg for No CKC; 134.0 ± 3.6 μg for 25 mM CKC; 141.7 ± 14.4 μg for 100 mM CKC; 130.0 ± 8.7 μg for 200 mM CKC. Data are presented as mean ± standard deviation with *n* = 3.

The in vitro release data for NPNa are shown in Figure 7. The control and 25 mM CKC contact lenses release 70% of NPNa in only 4 h. The release time increases for 100 mM CKC contact lenses, releasing 70% of NPNa in approximately 12 h. Finally, 200 mM CKC contact lenses release 70% of NPNa after 25 h compared to 4 h for the control contact lenses. For NPNa, the increase in release duration becomes more apparent for ≥100 mM CKC concentration (see Figure 7), which corresponds to formation of aggregates as observed by shear thinning behavior at this CKC concentration range (see Figure 2).

Note that by utilizing CKC, the extent of the increase in release duration for NPNa is not as significant as corresponding ones for DFNa and FBNa. As discussed in Section 3.1, DFNa forms viscous aggregates at lower CKC concentrations compared to NPNa due to its greater hydrophobicity (i.e., log *P*). Among the three drugs studied, DFNa has the highest log *P*, while NPNa has the lowest log *P* (Table 1). The observed correlation between the rheology results, log *P*, and the release kinetic studies suggests that a drug with a higher log *P* forms viscoelastic catanionic aggregates more easily.

Several studies on drug delivery from contact lenses have modeled the drug release as a function of time [3,18,38,59]. Since the diameter of a lens is much larger than its thickness, the geometry of a lens can be modeled as thin flat film with variable thickness [3]. Thus, the following equation can be used to predict the cumulative drug percent release from a contact lens as a function of time [3,18,38,59]:

(5) $$\frac{M_{t}}{M_{\infty }}=\;1-\overset{\infty }{\underset{n=0}{\sum }}\frac{8}{{\left(2n+1\right)}^{2}\pi^{2}}\exp\left[-\frac{{\left(2n+1\right)}^{2}\pi^{2}Dt}{4h^{2}}\;\right],$$

where *h* is the half-thickness of the contact lens, *D* is the effective drug diffusivity, and $M_{\infty }$ is the amount of drug released at an infinite time. Equation (5) is plotted in Figure 5, Figure 6, and Figure 7 as solid lines to compare the experimental drug release data with the corresponding predicted data. A good agreement between the model and experimental drug release data is observed.

**Figure 8:**
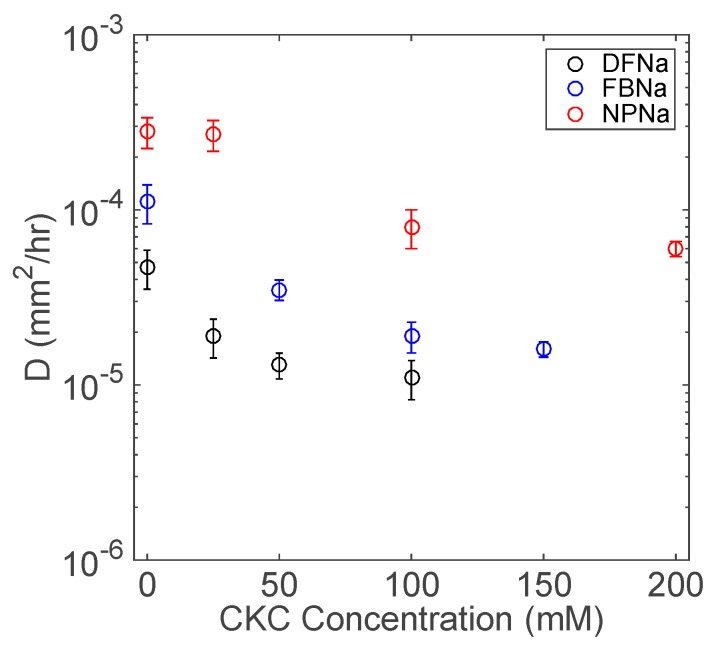
Fitted values of drug diffusivities at determined CKC concentrations for each of the three tested drugs. Data are presented as mean ± standard deviation with *n* = 3.

From Equation (5), *D* the effective diffusivity from contact lenses is estimated from the experimental drug release data. Figure 8 shows the estimated effective diffusivities as a function of CKC concentration for the NSAIDs studied in this paper. For all drugs, as the CKC concentration increases, we observe an asymptotic decay in the effective diffusivity profiles. For instance, at 25 or 50 mM CKC in DFNa solution, the drug diffusivity significantly decreases, whereas by a further increase of CKC concentration to 100 mM, the decrease in the effective diffusivity is less significant. Thus, further extension of release duration is expected to be insignificant for CKC concentrations above 100 mM.

### 3.3. Characterization of Contact Lenses

**Figure 9:**
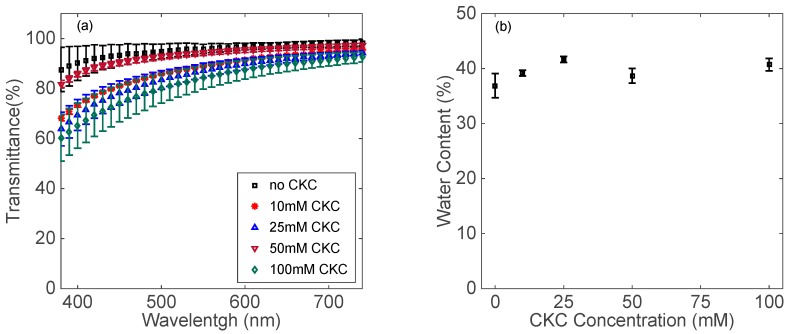
(**a**) Optical transmission of contact lenses embedded with catanionic aggregates between CKC and diclofenac sodium. (**b**) Water content of contact lenses embedded with catanionic aggregates between CKC and diclofenac sodium. Data are presented as mean ± standard deviation with *n* = 3.

Contact lenses have important properties, e.g., optical transparency and water content, which should not be compromised with the incorporation of aggregates or any type of nanoparticles. Also, the oxygen permeability [60,61] and material modulus [62] are associated with the lens water content. An acceptable range for optical transparency of contact lenses is usually around 90% [63]. As shown in Figure 9a, the optical clarity of contact lenses decreases by increasing the CKC concentration, yet remains close to the optimal clarity range. By increasing CKC concentration, water content is slightly increased by about 5–10% (see Figure 9b) and is not expected to have an adverse impact on the contact lens critical properties.

## 4. Conclusions

The present work focused on extending the release duration of three NSAIDs drugs, namely DFNa, FBNa, and NPNa, from pHEMA contact lenses. It was shown that the release time can be extended with the formation of drug-participating catanionic aggregates. These aggregates are formed due to the ionic and hydrophobic interactions between a long-alkyl chain cationic surfactant and an anionic drug in aqueous solutions. Steady and dynamic shear rheology showed that highly viscous catanionic aggregates present shear thinning and viscoelastic behavior typical of wormlike micelles, depending on the drug type and CKC concentration. For instance, more hydrophobic drugs such as DFNa showed stronger effects on promoting the formation of viscoelastic catanionic aggregates than less hydrophobic drugs, e.g., NPNa. Furthermore, the Maxwellian fluid model, which represents solutions with wormlike micelles, showed good agreement with the obtained experimental data. We showed that the increase in the release time depends on three factors: (i) the concentration of the cationic surfactant, (ii) the alkyl chain length of the cationic surfactant, and (iii) the octanol–water partition coefficient of each NSAID.

It is believed that the mechanism of extended release is due to the entrapment of the drugs in viscoelastic catanionic aggregates, which retards the diffusivity of drugs to the release medium. For all studied drugs, by increasing the CKC concentration, the release duration was extended. Furthermore, C8, which has a shorter alkyl chain length than CKC, was unable to form drug-participating catanionic aggregates. Thus, the release extension for C8-based contact lenses was insignificant. The release time of drugs with higher log *P* values, e.g., DFNa, was enhanced in duration in comparison to drugs with smaller log *P* values such as NPNa. Finally, it was shown that the transmittance and water content of the contact lenses were not significantly compromised by the addition of CKC. Future studies should involve the use of small-angle scattering and transmission electron microscopy that can help in the confirmation of the wormlike morphology of the micelles and in the characterization of their size. Additionally, these studies should evaluate the material properties of the contact lenses, such as oxygen permeability, material modulus, and wettability. Biocompatibility studies should also be performed to assess any potential risk of cytotoxicity before proceeding to in vivo studies.

## Figures and Tables

**Table 1:** Physicochemical properties of the three model drugs (source: ALOGPS from DrugBank).

Drug	Molar Mass (g/mol)	Physiological Charge	Log *P*
Diclofenac sodium	318	−1	4.75
Flurbiprofen sodium	266	−1	4.07
Naproxen sodium	252	−1	3.39

## References

[1] Bachu R., Chowdhury P., Al-Saedi Z., Karla P., Boddu S. (2018). Ocular Drug Delivery Barriers—Role of Nanocarriers in the Treatment of Anterior Segment Ocular Diseases. Pharmaceutics.

[2] Le Bourlais C., Acar L., Zia H., Sado P.A., Needham T., Leverge R. (1998). Ophthalmic drug delivery systems—Recent advances. Prog. Retin. Eye Res..

[3] Peng C.-C., Kim J., Chauhan A. (2010). Extended delivery of hydrophilic drugs from silicone-hydrogel contact lenses containing Vitamin E diffusion barriers. Biomaterials.

[4] Kim J., Peng C.-C., Chauhan A. (2010). Extended release of dexamethasone from silicone-hydrogel contact lenses containing vitamin E. J. Control. Release.

[5] Alvarez-Lorenzo C., Hiratani H., Gómez-Amoza J.L., Martínez-Pacheco R., Souto C., Concheiro A. (2002). Soft Contact Lenses Capable of Sustained Delivery of Timolol. J. Pharm. Sci..

[6] Ciolino J.B., Stefanescu C.F., Ross A.E., Salvador-Culla B., Cortez P., Ford E.M., Wymbs K.A., Sprague S.L., Mascoop D.R., Rudina S.S. (2014). In vivo performance of a drug-eluting contact lens to treat glaucoma for a month. Biomaterials.

[7] Paradiso P., Serro A.P., Saramago B., Colaço R., Chauhan A. (2016). Controlled Release of Antibiotics from Vitamin E–Loaded Silicone-Hydrogel Contact Lenses. J. Pharm. Sci..

[8] Peng C.-C., Burke M.T., Carbia B.E., Plummer C., Chauhan A. (2012). Extended drug delivery by contact lenses for glaucoma therapy. J. Controll. Release.

[9] Gulsen D., Chauhan A. (2005). Dispersion of microemulsion drops in HEMA hydrogel: A potential ophthalmic drug delivery vehicle. Int. J. Pharm..

[10] Torres-Luna C., Hu N., Koolivand A., Fan X., Zhu Y., Domszy R., Yang J., Yang A., Wang N.S. (2019). Effect of a Cationic Surfactant on Microemulsion Globules and Drug Release from Hydrogel Contact Lenses. Pharmaceutics.

[11] Li C.-C., Abrahamson M., Kapoor Y., Chauhan A. (2007). Timolol transport from microemulsions trapped in HEMA gels. J. Colloid Interface Sci..

[12] Kapoor Y., Chauhan A. (2008). Ophthalmic delivery of Cyclosporine A from Brij-97 microemulsion and surfactant-laden p-HEMA hydrogels. Int. J. Pharm..

[13] Maulvi F.A., Mangukiya M.A., Patel P.A., Vaidya R.J., Koli A.R., Ranch K.M., Shah D.O. (2016). Extended release of ketotifen from silica shell nanoparticle-laden hydrogel contact lenses: In vitro and in vivo evaluation. J. Mater. Sci. Mater. Med..

[14] Maulvi F.A., Desai A.R., Choksi H.H., Patil R.J., Ranch K.M., Vyas B.A., Shah D.O. (2017). Effect of surfactant chain length on drug release kinetics from microemulsion-laden contact lenses. Int. J. Pharm..

[15] Hiratani H., Alvarez-Lorenzo C. (2002). Timolol uptake and release by imprinted soft contact lenses made of N,N-diethylacrylamide and methacrylic acid. J. Controll. Release.

[16] Torres-Luna C., Hu N., Tammareddy T., Domszy R., Yang J., Wang N.S., Yang A. (2019). Extended delivery of non-steroidal anti-inflammatory drugs through contact lenses loaded with Vitamin E and cationic surfactants. Contact Lens Anterior Eye.

[17] Sekar P., Chauhan A. (2019). Effect of vitamin-E integration on delivery of prostaglandin analogs from therapeutic lenses. J. Colloid Interface Sci..

[18] Dixon P., Chauhan A. (2017). Effect of the surface layer on drug release from delefilcon-A (Dailies Total1) contact lenses. Int. J. Pharm..

[19] Hsu K.H., Lazon de la Jara P., Ariyavidana A., Watling J., Holden B., Garrett Q., Chauhan A. (2015). Release of Betaine and Dexpanthenol from Vitamin E Modified Silicone-Hydrogel Contact Lenses. Curr. Eye Res..

[20] Dixon P., Fentzke R.C., Bhattacharya A., Konar A., Hazra S., Chauhan A. (2018). In vitro drug release and in vivo safety of vitamin E and cysteamine loaded contact lenses. Int. J. Pharm..

[21] Ciolino J.B., Hoare T.R., Iwata N.G., Behlau I., Dohlman C.H., Langer R., Kohane D.S. (2009). A Drug-Eluting Contact Lens. Investig. Opthalmol. Vis. Sci..

[22] Pall B., Gomes P., Yi F., Torkildsen G. (2019). Management of Ocular Allergy Itch with an Antihistamine-Releasing Contact Lens. Cornea.

[23] ClinicalTrials.gov Bethesda (MD): National Library of Medicine (US). 18 January 2018–August 2019. Identifier NCT02852057, Effectiveness and Safety of Timolol and Dorzolamide Loaded Contact Lenses. NCT02852057.

[24] Dew N., Bramer T., Edsman K. (2008). Catanionic aggregates formed from drugs and lauric or capric acids enable prolonged release from gels. J. Colloid Interface Sci..

[25] Bramer T., Paulsson M., Edwards K., Edsman K. (2003). Catanionic Drug–Surfactant Mixtures: Phase Behavior and Sustained Release from Gels. Pharm. Res..

[26] Bramer T., Dew N., Edsman K. (2006). Catanionic mixtures involving a drug: A rather general concept that can be utilized for prolonged drug release from gels. J. Pharm. Sci..

[27] Jiang Y., Luan Y., Qin F., Zhao L., Li Z. (2012). Catanionic vesicles from an amphiphilic prodrug molecule: A new concept for drug delivery systems. RSC Adv..

[28] Paulsson M., Edsman K. (2001). Controlled Drug Release from Gels Using Surfactant Aggregates. II. Vesicles Formed from Mixtures of Amphiphilic Drugs and Oppositely Charged Surfactants. Pharm. Res..

[29] Jiang Y., Li F., Luan Y., Cao W., Ji X., Zhao L., Zhang L., Li Z. (2012). Formation of drug/surfactant catanionic vesicles and their application in sustained drug release. Int. J. Pharm..

[30] Dew N., Edwards K., Edsman K. (2009). Gel formation in systems composed of drug containing catanionic vesicles and oppositely charged hydrophobically modified polymer. Colloids Surf. B Biointerfaces.

[31] Dew N., Edsman K., Björk E. (2011). Novel gel formulations with catanionic aggregates enable prolonged drug release and reduced skin permeation: Gel formulations with catanionic aggregates. J. Pharm. Pharmacol..

[32] Bramer T., Dew N., Edsman K. (2007). Pharmaceutical applications for catanionic mixtures. J. Pharm. Pharmacol..

[33] Ji X., Shi C., Qi L., Guo Y., Li N., Li Z., Luan Y. (2014). Preparation, properties and in vivo pharmacokinetic study of drug vesicles composed of diphenhydramine and AOT. RSC Adv..

[34] Shields C.W., Wang L.L., Evans M.A., Mitragotri S. (2019). Materials for Immunotherapy. Adv. Mater..

[35] Jiang Y., Hu X., Zhang J., Jin G., Luan Y. (2019). Chlorambucil prodrug-participating catanionic aggregates for sustained drug release and improved antitumour activity. J. Mol. Liq..

[36] Kapoor Y., Chauhan A. (2008). Drug and surfactant transport in Cyclosporine A and Brij 98 laden p-HEMA hydrogels. J. Colloid Interface Sci..

[37] Kapoor Y., Thomas J.C., Tan G., John V.T., Chauhan A. (2009). Surfactant-laden soft contact lenses for extended delivery of ophthalmic drugs. Biomaterials.

[38] Bengani L.C., Chauhan A. (2013). Extended delivery of an anionic drug by contact lens loaded with a cationic surfactant. Biomaterials.

[39] Diestelhorst M., Schmidl B., Konen W., Mester U., Sunder Raj P. (1996). Efficacy and tolerance of diclofenac sodium 0.1%, flurbiprofen 0.03%, and indomethacin 1.0% in controlling postoperative inflammation. J. Cataract Refract. Surg..

[40] Zhou Y., Cho K.-J., Plowman S.J., Hancock J.F. (2012). Nonsteroidal Anti-inflammatory Drugs Alter the Spatiotemporal Organization of Ras Proteins on the Plasma Membrane. J. Biol. Chem..

[41] Boggara M.B., Krishnamoorti R. (2010). Partitioning of Nonsteroidal Antiinflammatory Drugs in Lipid Membranes: A Molecular Dynamics Simulation Study. Biophys. J..

[42] Liberatore M.W., Nettesheim F., Vasquez P.A., Helgeson M.E., Wagner N.J., Kaler E.W., Cook L.P., Porcar L., Hu Y.T. (2009). Microstructure and shear rheology of entangled wormlike micelles in solution. J. Rheol..

[43] Raghavan S.R., Edlund H., Kaler E.W. (2002). Cloud-Point Phenomena in Wormlike Micellar Systems Containing Cationic Surfactant and Salt. Langmuir.

[44] Kim W.-J., Yang S.-M. (2000). Effects of Sodium Salicylate on the Microstructure of an Aqueous Micellar Solution and Its Rheological Responses. J. Colloid Interface Sci..

[45] Bijma K., Engberts J.B.F.N. (1997). Effect of Counterions on Properties of Micelles Formed by Alkylpyridinium Surfactants. 1. Conductometry and ^1^ H-NMR Chemical Shifts. Langmuir.

[46] Bijma K., Rank E., Engberts J.B.F.N. (1998). Effect of Counterion Structure on Micellar Growth of Alkylpyridinium Surfactants in Aqueous Solution. J. Colloid Interface Sci..

[47] Kumar R., Kalur G.C., Ziserman L., Danino D., Raghavan S.R. (2007). Wormlike Micelles of a C22-Tailed Zwitterionic Betaine Surfactant: From Viscoelastic Solutions to Elastic Gels. Langmuir.

[48] Davies T.S., Ketner A.M., Raghavan S.R. (2006). Self-Assembly of Surfactant Vesicles that Transform into Viscoelastic Wormlike Micelles upon Heating. J. Am. Chem. Soc..

[49] Larson R.G. (1999). Structure and Rheology of Complex Fluids.

[50] Raghavan S.R., Fritz G., Kaler E.W. (2002). Wormlike Micelles Formed by Synergistic Self-Assembly in Mixtures of Anionic and Cationic Surfactants. Langmuir.

[51] Raghavan S.R., Kaler E.W. (2001). Highly Viscoelastic Wormlike Micellar Solutions Formed by Cationic Surfactants with Long Unsaturated Tails. Langmuir.

[52] Hsu K.-H., Gupta K., Nayaka H., Donthi A., Kaul S., Chauhan A. (2017). Multidose Preservative Free Eyedrops by Selective Removal of Benzalkonium Chloride from Ocular Formulations. Pharm. Res..

[53] Sharma R., Mahajan R.K. (2012). An investigation of binding ability of ionic surfactants with trifluoperazine dihydrochloride: Insights from surface tension, electronic absorption and fluorescence measurements. RSC Adv..

[54] Singh O., Kaur R., Aswal V.K., Mahajan R.K. (2016). Composition and Concentration Gradient Induced Structural Transition from Micelles to Vesicles in the Mixed System of Ionic Liquid–Diclofenac Sodium. Langmuir.

[55] Vaid Z.S., Kumar A., El Seoud O.A., Malek N.I. (2017). Drug induced micelle-to-vesicle transition in aqueous solutions of cationic surfactants. RSC Adv..

[56] Rajput S.M., Gangele K., Kumar S., Aswal V.K., Mata J.P., Malek N.I. (2018). Nano-Vehicles for Drug Delivery Using Low-Cost Cationic Surfactants: A Drug Induced Structural Transitions. ChemistrySelect.

[57] Zhao L., Liu J., Zhang L., Gao Y., Zhang Z., Luan Y. (2013). Self-assembly properties, aggregation behavior and prospective application for sustained drug delivery of a drug-participating catanionic system. Int. J. Pharm..

[58] Avdeef A., Box K.J., Comer J.E., Hibbert C., Tam K.Y. (1998). Determination of liposomal membrane water partition coefficients of ionizable drugs. Pharm. Res..

[59] Silva A.S.G., Pinheiro M.N.C. (2013). Diffusion Coefficients of Timolol Maleate in Polymeric Membranes Based on Methacrylate Hydrogels. J. Chem. Eng. Data.

[60] Willis S.L., Court J.L., Redman R.P., Wang J.H., Leppard S.W., O’Byrne V.J., Small S.A., Lewis A.L., Jones S.A., Stratford P.W. (2001). A novel phosphorylcholine-coated contact lens for extended wear use. Biomaterials.

[61] Holden D.A., Merrz G.W. (1984). Critical Oxygen Levels to Avoid Corneol Edema for Daily and Extended Wear Contact Lenses. Investig. Ophthalmol. Vis. Sci..

[62] Horst C.R., Brodland B., Jones L.W., Brodland G.W. (2012). Measuring the Modulus of Silicone Hydrogel Contact Lenses. Optom. Vis. Sci..

[63] Guzman-Aranguez A., Colligris B., Pintor J. (2013). Contact Lenses: Promising Devices for Ocular Drug Delivery. J. Ocul. Pharmacol. Ther..

